# Silver Nanoparticles for Fluorescent Nanocomposites by High-Pressure Magnetron Sputtering

**DOI:** 10.3390/ma16041591

**Published:** 2023-02-14

**Authors:** Tomáš Zikmund, Jiří Bulíř, Michal Novotný, Ladislav Fekete, Sergii Chertopalov, Stefan Andrei Irimiciuc, Mariana Klementová, Jarmila Balogová, Jan Lančok

**Affiliations:** 1 FZU—Institute of Physics of the Czech Academy of Sciences, Na Slovance 2, 182 00 Prague, Czech Republic; 2 Faculty of Nuclear Sciences and Physical Engineering, Czech Technical University, Brehova 7, 115 19 Prague, Czech Republic; 3 National Institute for Lasers Plasma and Radiation Physics, Atomistilor 409, 077125 Magurele, Romania

**Keywords:** Ag nanoparticles, surface plasmon resonance, photoluminescence

## Abstract

We report on the formation of silver nanoparticles by gas aggregation in a reaction chamber at room temperature. The size distribution of nanoparticles deposited on a silicon substrate for various lengths of an aggregation (high-pressure) chamber was investigated by atomic force microscopy. Nanoparticles were characterized by scanning and transmission electron microscopy and spectral ellipsometry. The physical shape of the nanoparticles and its distribution was correlated with their optical properties. Metal–dielectric nanocomposites were deposited employing simultaneous deposition of Ag NPs via high-pressure magnetron sputtering and the dielectric matrix was deposited via thermal evaporation. Pure and Eu-, Er-, and Yb-doped lithium fluoride was used as the dielectric host matrix. Optical transmittance of lithium fluoride containing silver nanoparticles was measured and their theoretical absorption cross-section calculated. The nanoparticles were also embedded in Eu^3+^-doped downshifting and Er^3+^- and Y^b3+^-doped up-conversion materials to study their influence on emission spectra. Spectra of identical layers with and without nanoparticles were compared. Their transmittance at various annealing temperatures is also presented.

## 1. Introduction

Apart from widely used chemical methods for preparing nanoparticles (NPs), many physical methods for metal nanoparticle fabrication have already been developed as well [1]. These range from very low-priced, large scale industrial methods such as ball milling [2] through very slow and precise lithographic methods to vacuum deposition methods such as arc discharge or pulse laser deposition. Physical methods for nanoparticle formation are generally based on the gas aggregation method, which utilizes condensation of saturated vapor in a chamber at elevated pressure. The pressure usually ranges from tens to hundreds of Pa. Initial gas aggregation sources (GAS) used a heated evaporation crucible [3] as a vapor source. A GAS using magnetron sputtering was first introduced by H. Haberland [4] in 1992. Over time, many variations of this source have been developed and successfully implemented for various applications [5,6]. High-pressure magnetron sputtering (HPMS) was shown to be a versatile bottom-up method with precise control over the manufacturing process for direct deposition of NPs [7].

Silver (Ag) NPs are known to have a strong plasmonic response in the visible to infrared region and have a plethora of industrial and technological applications. Their properties, especially size and shape, can be controlled by HPMS discharge parameters such as pressure, temperature, gas flow, size, etc. [8]. Alternatively, nanocomposites containing dispersed metallic nanoparticles have also attracted a great deal of interest in recent years. Their unique optical, electronic, and magnetic properties significantly enhance the performance of devices such as light-emitting diodes (LEDs) [9], photovoltaic solar cells [10,11], optical isolators [12], and gas sensors [13].

In this paper, we report on the formation of silver nanoparticles in a high-pressure aggregation chamber. We show clear dependencies between the mean particle size and the length of the aggregation chamber. Ag nanoparticles were deposited on a silicon substrate with the magnetron sputtering head in various positions, and the particle-size distribution was measured via atomic force microscopy (AFM). The optical properties were investigated by immersing the Ag NP in a LiF matrix via simultaneous co-deposition. Ag NP influence on lanthanide-ion downshifting and up-conversion photoemission was also investigated by embedding these in Eu^3+^-doped LaF3 and in Er^3+^- and Yb^3+^-doped LaF_3_. Lanthanum fluoride is known to be a good host matrix for efficient lanthanide ion photoemission [14,15] and has an important advantage, as it can be evaporated congruently with various stoichiometries from a single crucible [16,17]. Eu^3+^ was chosen because its main absorption peak at 395 nm overlaps with the SPR frequency of Ag NPs produced by HPMS in our setup. A key factor in our approach was to elucidate whether the energy absorbed by Ag NPs can be transferred to Eu^3+^ ions to increase their emission intensity. We also compared the spectral shape of Eu^3+^-doped LaF_3_ with and without Ag NPs.

## 2. Materials and Methods

### 2.1. Deposition Setup

**Figure 1:**
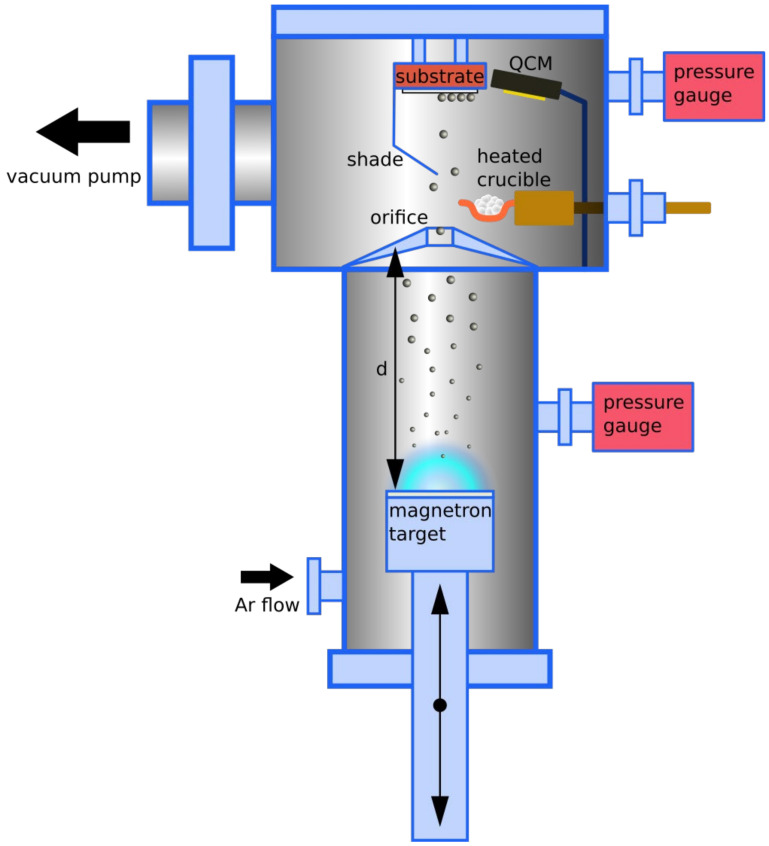
A schematic representation of the deposition apparatus.

All the samples were prepared in a deposition chamber consisting of an aggregation high-pressure chamber with a magnetron head and a main chamber with a thermal evaporation source. The two sections of the chamber were separated by an orifice with a diameter of 4 mm. The pressure in the aggregation chamber was adjusted via argon (Ar) flow. Ag atoms were sputtered from a magnetron target via plasma discharge and traveled through the aggregation chamber to the orifice, where they entered the main chamber. A 2-inch silver target (99.99%) was connected to a DC power supply. The sputtering power was set to 50 W for all the samples. In the aggregation chamber, the Ag atoms collided and aggregated into larger clusters and nanoparticles (NPs) that passed through the orifice to the main chamber. A heated molybdenum evaporation boat placed in the main chamber enabled the simultaneous deposition of a dielectric matrix material and NPs on a substrate positioned in front of the orifice and the heated crucible. A shield was used to block approximately 50% of the of the NP flow, so that the NPs were only dispersed on one half of the substrate area, while the dielectric material covered the whole area of the substrate. This approach allowed us to compare properties of the resulting layer with and without NPs. A schematic representation of the deposition setup is presented in Figure 1.

### 2.2. Production and Analysis of Ag Nanoparticles and Nanocomposite Materials

Ag NPs were deposited on a p-doped silicon substrate without any dielectric matrix in order to measure the size distribution at variable target–orifice distances. The deposition time was 40 s. The size analysis was performed via nanoparticle height measurement using AFM (Bruker Dimension Icon, Billerica, USA) in peak-force tapping mode. Scanning electron microscopy (SEM, Tescan Fera3, Brno, Czech Republic) images were taken to estimate the lateral dimensions of nanoparticles. Spectral ellipsometry measurement was performed in order to estimate the optical properties of the effective media.

A series of Ag NPs in LiF-matrix nanocomposite samples were prepared for optical transmittance measurements. The LiF/Ag nanocomposite films were deposited via simultaneous deposition of LiF (Sigma-Aldrich, St. Louis, USA, powder 99.99%) from a heated boat and of Ag NPs via HPMS for various target–orifice distances. The pressure in the aggregation chamber was set to 30 Pa using a 25 sccm flow of Ar while the pressure in the main chamber was approximately 1.2 Pa. The deposition time ranged from 1 to 2 min. Nanocomposite materials were deposited on a glass substrate heated to 200 °C. The resulting thicknesses of the nanocomposite layers were in the 100 to 200 nm range. A 60 nm protective coating of pure LiF was deposited on the top in order to prevent contamination of the deposited samples.

A special sample was deposited on a NaCl substrate for transmission electron microscopy (TEM) measurement. A 15 nm layer of CaF_2_ (Sigma-Aldrich, St. Louis, USA, 99.99%) was deposited, followed by a layer of Ag NPs (30 s deposition at 25 Pa). The resulting materials were covered by another 16 nm layer of CaF_2_. The CaF_2_ layer was deposited via evaporation of powder from a molybdenum boat. The Ag NPs were prepared with a target–orifice distance of 60 mm. The sample was then placed on a wet surface while the NaCl substrate was diluted, and the floating layer was extracted by a copper grid. TEM measurements were performed on an FEI Tecnai TF20 X-twin microscope (FEI, Eindhoven, The Netherlands) operated at 200 kV (FEG, 1.9 Å point resolution) with an EDAX energy-dispersive X-ray (EDX) detector attached. The high-resolution (HRTEM) images were recorded on a Gatan UltraScan 1000XP CCD camera (Gatan, Pleasanton, USA) with a resolution of 2048 × 2048 pixels using the Digital Micrograph software (version 3.42.3055.00) package. Images were also acquired in scanning mode (STEM) using a high-angle annular dark field (HAADF) detector, which is sensitive to the atomic number (the heavier elements appear brighter). The EDX maps were recorded in scanning TEM (STEM) mode using the TIA software (FEI, Eindhoven, The Netherlands). The maps were calculated via the full quantification method using AgL, FK, CaK, and OK lines.

Two classes of samples were fabricated to test the effect of Ag NPs on luminescence emission: a Eu^3+^-doped LaF_3_ nanocomposite for the down-shifting emission spectrum and a Yb^3+^: Er^3+^:LaF_3_ nanocomposite for the up-conversion emission spectrum. The nanocomposite containing Ag NPs and LaF_3_ doped with 15 at.% Eu^3+^ was deposited from a LaF_3_ (Sigma-Aldrich, St. Louis, USA, 99.99%) and EuF_3_ (Sigma-Aldrich, St. Louis, USA, 99.99%) mixed powder, which was evaporated from a molybdenum boat with simultaneous deposition of Ag NPs via HPMS. The distance between the target and orifice was 60 mm for all the deposited samples. The pressure in the aggregation chamber was set to 35 Pa using a 31 sccm flow of Ar. The pressure in the main chamber was approximately 1.5 Pa while the deposition time ranged from 1 to 3 min. The nanocomposite was deposited on a fused silica (FS) substrate at room temperature (22 °C). The thickness of the nanocomposite layer ranged from 500 to 1000 nm with a 200 to 300 nm cover layer without NPs.

The identical procedure was implemented for depositing the up-conversion nanocomposite. The fluorescent material was LaF_3_ doped with 7 at.% Yb^3+^ and 1 at.% Er^3+^. A mixture of LaF_3_, YbF_3_ (Sigma-Aldrich, 99.99%) and ErF_3_ (Sigma-Aldrich, 99.99%) powder was used for evaporation.

## 3. Results and Discussion

NP aggregation in high-pressure reaction chambers is, in general, conditioned by a large number of variables. Thus, the size of nanoparticles can depend on many parameters such as the pressure, composition [18,19,20], and temperature [21] of the working gas in the aggregation chamber. Other factors include magnetron power [19,22,23] and the distance between the magnetron target and the orifice [20,24].

**Figure 2:**
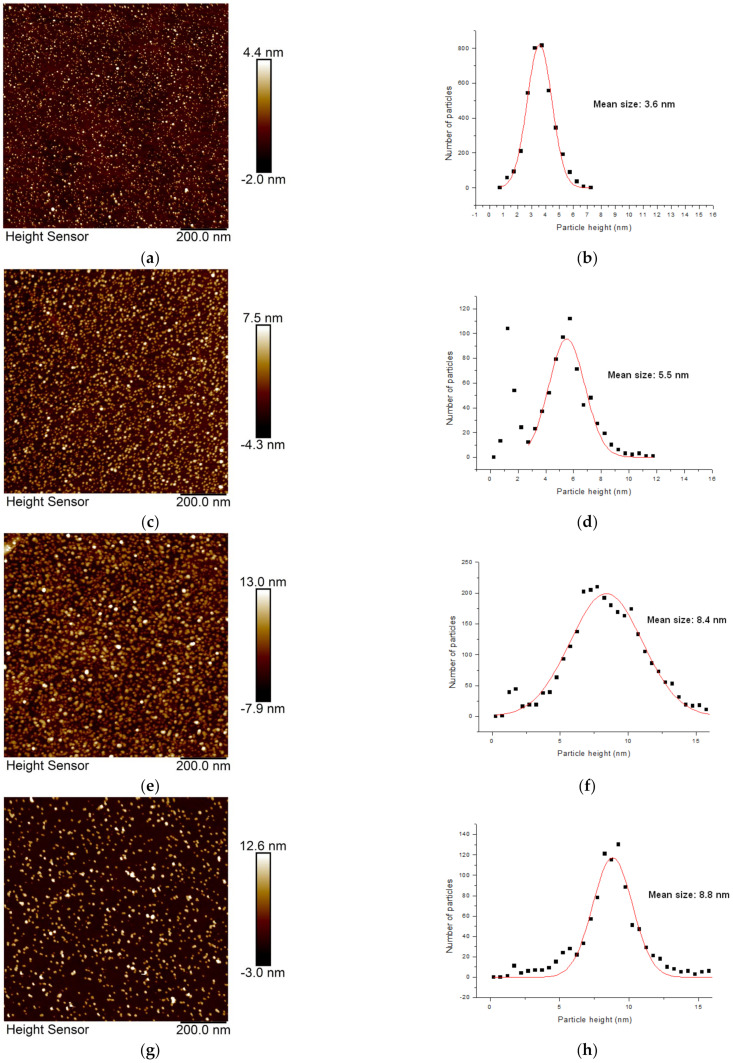
AFM images of Ag NPs deposited for various target–orifice distances: (**a**) 50 mm, (**c**) 80 mm, (**e**) 150 mm, and (**g**) 200 mm. The graphs show the size (height) distributions of the Ag NPs for the corresponding AFM images: (**b**), (**d**), (**f**), and (**h**), respectively.

The Ar pressure in the aggregation chamber can be set as a balance between the gas flow and the orifice diameter. For the reaction chamber used in this study, according to our measurements, no discernible dependence between NP size and the gas pressure was found. This result contradicts previous studies [18], where NP size reportedly increases with pressure. Because a higher pressure also means higher Ar flow, the NPs spent less time in the aggregation chamber. A shorter time can thus compensate for the effect of increased pressure. Furthermore, different magnetron powers (50 W and 100 W) were tested, with no significant effect on the properties of the resulting Ag NP. However, it was found that the size of the Ag NPs increases with the distance between the magnetron target and the orifice. Nanoparticle sizes were measured with an AFM height sensor for target–orifice distances ranging from 50 to 250 mm. Examples of AFM measurements for distances of 50, 80, 150, 200 mm are shown in the Figure 2 along with the corresponding height size distributions. The target–orifice distance has a clear influence on NP sizes, as a 4-fold increase in distance leads to a 2.4x increase in average NP size.

**Figure 3:**
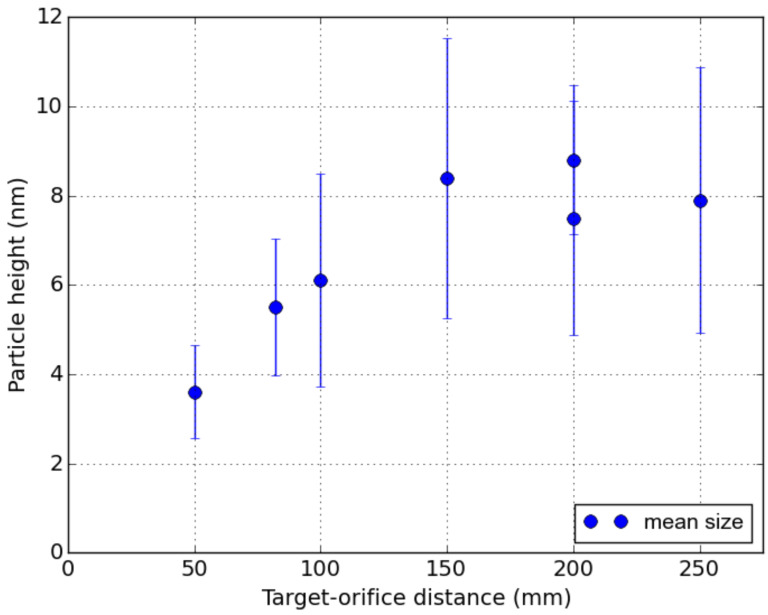
The mean height of nanoparticles versus the target–orifice distance. Error bars represent the FWHM of the size distribution.

The dependence of the average size of Ag NPs on the target–orifice distance is plotted in Figure 3. Error bars represent full-width half-maximum (FWHM) value of the size distribution. NP height increases with target–orifice distance, from about a mean size of 3.6 nm for 50 mm to about 8.4 nm for 150 mm. For longer distances (150 mm to 250 mm) the size remains constant at about 8 nm. These results are in partial agreement with previously published studies on metal nanoparticles [8,20,24]. The evolution of nanoparticle size, which is shown in Figure 3, corresponds to the aggregation process at high pressure. First, the Ag atoms are sputtered, forming a high concentration of Ag species in the gas near the sputtered target. Nucleation centers are formed due to supersaturation of the atoms at high pressure. The aggregated particles are carried by the gas stream, and their size gradually increases until the source of Ag atoms in the surrounding atmosphere is completely exhausted. Above that threshold point, the size of the aggregated nanoparticles does not increase. Cluster-size formation is mathematically described in [25]. It is clear from this theoretical work that the process of cluster formation has a threshold character.

**Figure 4:**
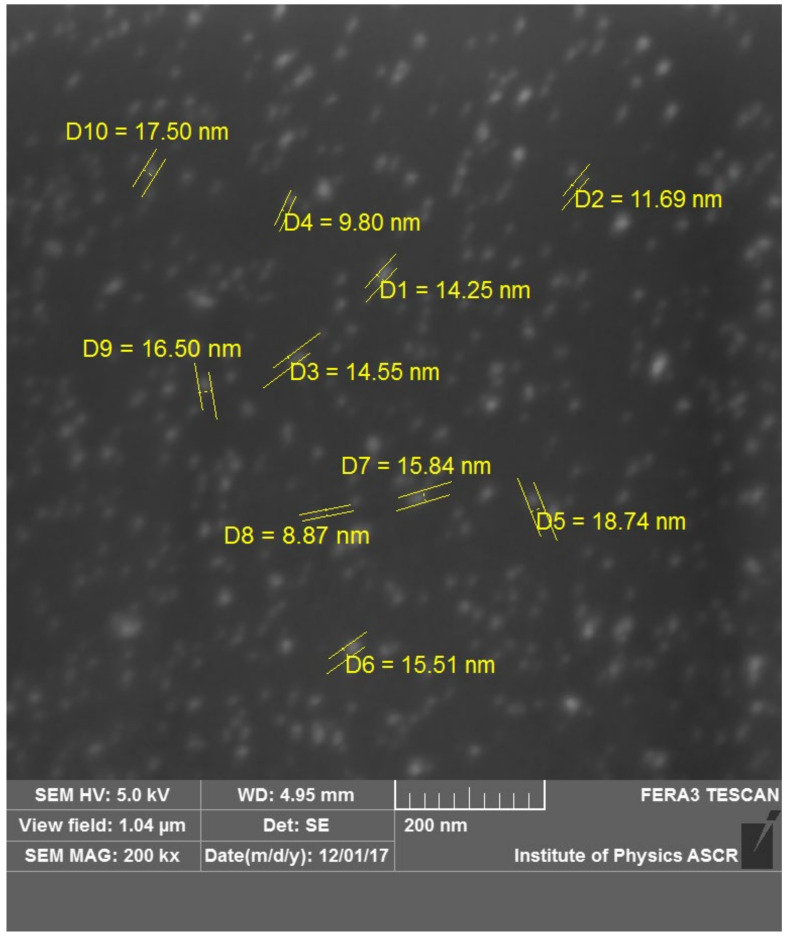
SEM image of Ag NPs on a Si substrate, deposited at a target–orifice distance of 200 mm.

The lateral diameter of Ag NPs was estimated via SEM. An image containing the approximate NP diameters of samples deposited at a target–orifice distance of 200 mm is presented in Figure 4. The mean NP size is around 14 nm, a value which is larger than the height measured by AFM. These differences are understandable as, due to their high impact speed, the NP can suffer deformation after reaching the substrate. Details on gas flow dynamics through an orifice are discussed in [26], where the Cu cluster drift velocity was determined to range from 90 ms^−1^ to 140 ms^−1^ for an Ar flow of 15 to 100 sccm through an orifice of diameter 3.5 mm.

**Figure 5:**
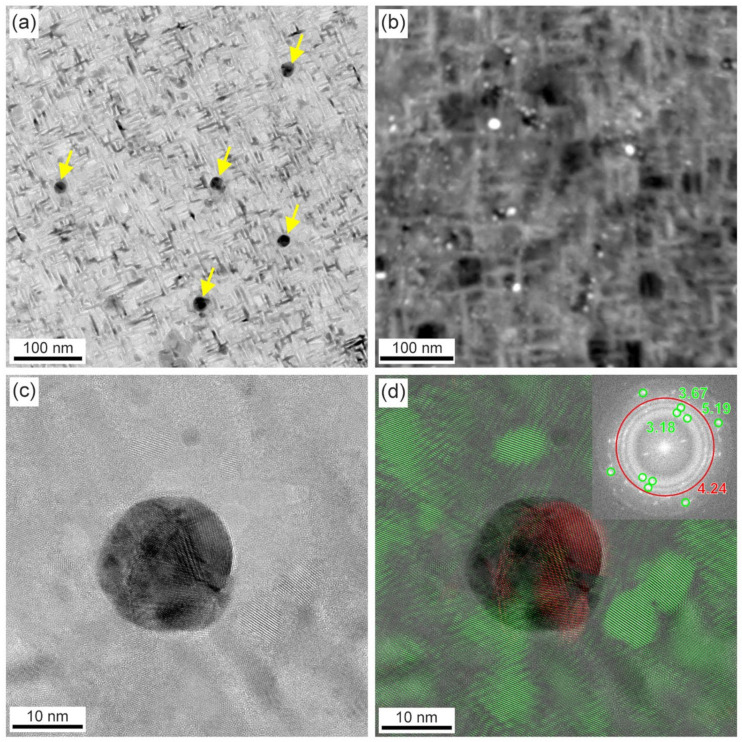
S/TEM results of the CaF_2_ containing Ag NPs deposited at a target–orifice distance of 60 mm. (**a**) Low-magnification BF–TEM image, (**b**) low magnification STEM/HAADF image, (**c**) HRTEM image of a single Ag NP showing its polycrystalline form, (**d**) HRTEM image overlaid with mapped CaF_2_ in green and cubic Ag in red (FFT inserted with the corresponding spatial frequencies marked).

**Figure 6:**
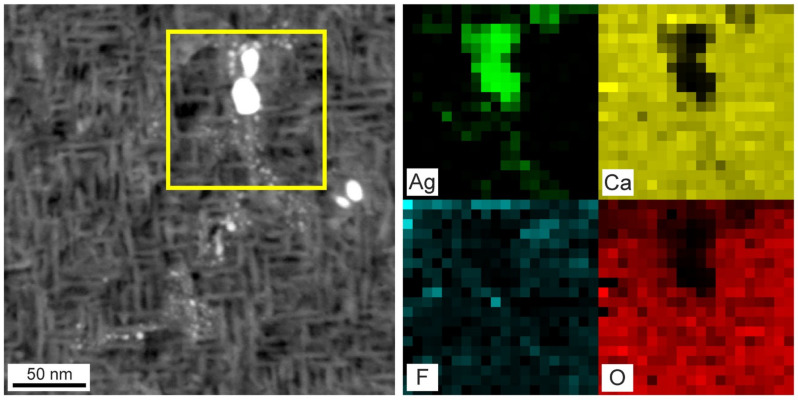
STEM (lefthand side)/EDX (righthand side) results showing the elemental composition analysis of Ag NPs in the CaF_2_ matrix. The yellow box frames the EDX analyzed sample.

Transmission electron microscopy results (TEM) of the 31 nm layer of CaF_2_ containing Ag NPs deposited at a target–orifice distance of 60 mm are shown in Figure 5 and Figure 6. In the TEM images, predominantly larger Ag NPs (10 to 20 nm in size) are discernible (Figure 5a). The smaller particles are likely to be obscured by the thick matrix and the strong diffraction contrast typical of TEM. However, in the STEM/HAADF images, a large number of smaller Ag NPs (a few nm in size) are clearly discernible (Figure 5b). This is due to the Z-contrast, which highlights the heavier elements with a contrast approximately proportional to Z^2^. Larger particles have a polycrystalline character as shown on the HRTEM image (Figure 5c), while the smaller ones are expected to be single crystals. The Ag NPs have cubic symmetry as confirmed by the fast-Fourier-transform (FFT) analysis performed on the HRTEM images. By filtering out the spatial frequencies of cubic Ag (d111 = 4.24 nm^−1^) and CaF_2_ (d022 = 5.19 nm^−1^, d002 = 3.67 nm^−1^, d111 = 3.18 nm^−1^), the corresponding phases were mapped on the images (Figure 5d). The distribution of phases aligns well with the image contrast and validates the cubic symmetry of Ag NPs. Moreover, the individual Ag NPs in the CaF_2_ matrix were mapped out by STEM/EDX (Figure 6). This confirms Ag inclusion in the NPs and also reveals a high concentration of oxygen in the CaF_2_ matrix. It is a very common phenomenon for fluorides to lose some fluorine during deposition in a non-fluorine atmosphere, where they can be replaced by oxygen.

**Figure 7:**
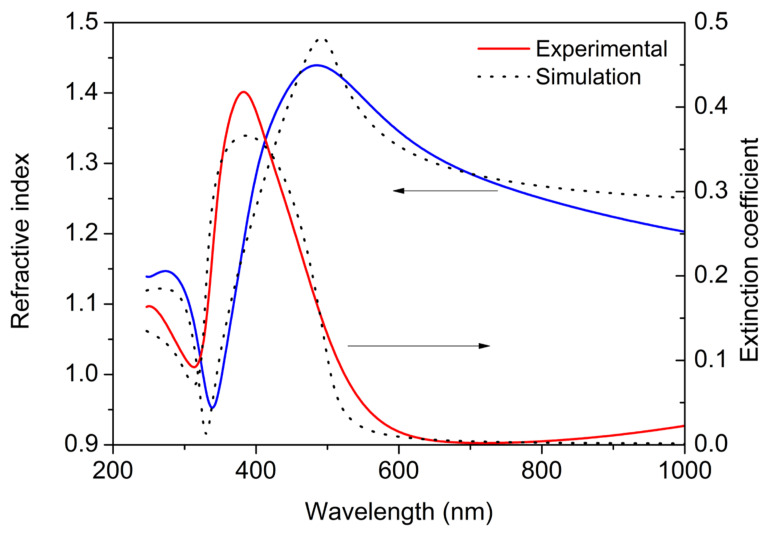
Refractive index (Blue line) and extinction coefficient (Red line) of Ag NPs estimated by spectral ellipsometry on a sample prepared on a Si substrate for a target–orifice distance of 60 mm. The dotted curve shows the simulation using Bruggeman model.

Optical functions, such as refractive index and extinction coefficient, represent the material properties that describe how the material interacts with light. A number of physical parameters can be derived from the spectral variation of optical functions. Figure 7 shows an estimated refractive index and extinction coefficient of Ag NPs as estimated by spectral ellipsometry. A 4-layer model (air/Ag NPs/silicon native oxide/silicon substrate) was used to simulate the measured ellipsometric data. The dispersion of the optical constants for the Ag NPs was described using a model using five Gaussian oscillators. The extinction coefficient peaks at approximately 380 nm, which corresponds to the localized surface plasmon resonance (LSPR) of Ag NPs, and decreases to approximately zero for longer wavelengths. The refractive index exhibits anomalous dispersion in the spectral range around the absorption peak, conserving the Kramers–Kronig relations. For longer wavelengths, it decreases to 1.15 at 1700 nm. The best fit thickness of the effective media was estimated 11 nm. There exists a correlation between geometrical features measured by AFM and SEM and the optical behavior of the Ag NPs [27,28]. The Ag NPs can be considered an effective media layer of a certain thickness. The refractive index and extinction coefficient functions obtained can be modeled using an effective medium approximation based on the mixing of two known components: silver and voids. The Bruggeman model is suitable for a multi-component system of effective media where the proportions of individual components are comparable [29]. By counting the Ag NPs on the SEM image (Figure 4), the proportion of Ag in the effective medium was estimated to be between 10 and 15%. We obtained a similar result when fitting the Bruggeman model to the measured optical functions, where the Ag fraction was estimated to be 14%. The simulation of the optical functions of the effective medium is shown in Figure 7 as a dotted curve. We can see some similarities, although the curves do not match each other perfectly. Obviously, the Bruggeman model has some limitations and should be applied with caution. For example, there is a limitation on the size of metal inclusions. Below a certain size limit, their dielectric functions cannot be assumed to be the same as in bulk due to the scattering of free electrons on the nanoparticle surface. In addition, the Bruggeman model does not take into account the geometry and shape of individual components. This is especially important for metal nanoparticles.

**Figure 8:**
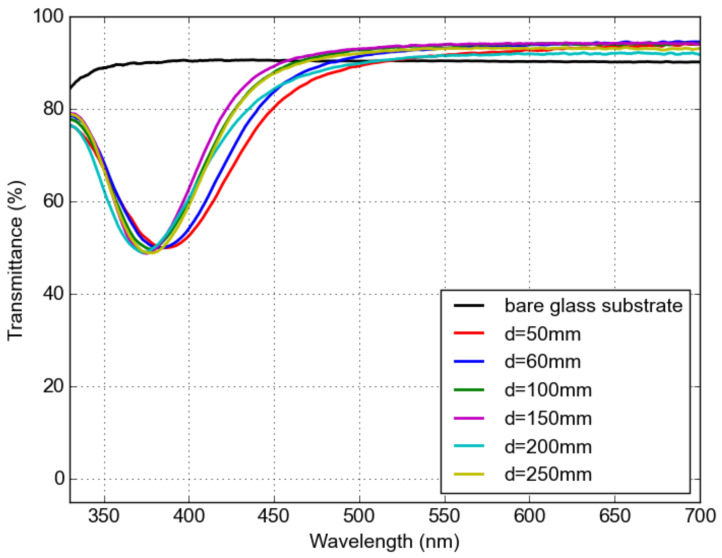
Optical transmission measurement of a LiF and Ag NP nanocomposite on a glass substrate for various target–orifice distances.

Similar deposition conditions were used for the preparation of Ag NPs in a LiF host medium. The Ag NPs induce an absorption peak, which can be observed in the visible range of the transmittance spectra. Transmission measurements of Ag NPs/ LiF nanocomposite layers prepared at various target–orifice distances are shown in Figure 8. Absorption peaks between 370 nm and 380 nm for all deposited samples. There is almost no difference among the samples, indicating that the NP size difference is too small to affect the LSPR peak position.

**Figure 9:**
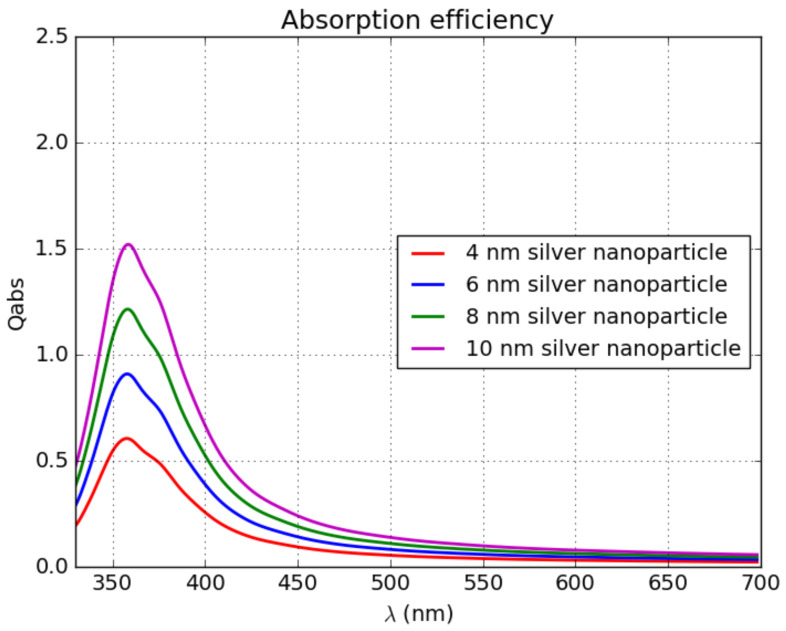
Mie calculation of absorption cross-section of Ag NP in LiF matrix.

Theoretical absorption cross-section spectra of silver NPs with diameters ranging from 4 to 10 nm was calculated using Mie theory [30] for comparison with the transmission measurement (compare Figure 9 versus Figure 8). LSPR absorption peaks at a wavelength of 355–360 nm, which is slightly shifted to a shorter wavelength compared to the measured data. For the Ag NPs that exhibit an oblate spheroid shape, different dipole modes of surface plasmon extinction can be observed. The split modes can present both a blue and a red spectral shift with respect to the classical Mie theory of spherical NPs [30]. No significant separation of the modes was observed in the recorded extinction spectra (Figure 7). The model has certain limitations, as it considers only a single nanoparticle without the influence of surrounding particles. It has already been proved that resonant coupling of neighboring NPs can shift the resulting resonance peak to a longer wavelength [31]. With certain approximations, the shift is inversely proportional to the gap between the neighboring NPs, which can explain the shift of the absorption peak in the measured data to the longer wavelengths. The observed variation in the position of the absorption peak may be influenced by the difference in NP density and the corresponding gap between neighboring NPs. An antireflection effect is observed in transmittance spectra for wavelengths higher than 500 nm, which is caused by the low refractive index of the effective media of Ag NPs in that spectral region.

Metal nanostructures can generally affect luminescence emission via three competing mechanisms. The first mechanism relates to fluorescence quenching in close proximity to the metal, which involves an energy transfer from an excited atom to the metal surface. The second relates to the significant electromagnetic field enhancement in the vicinity of the metal nanostructure via plasmonic resonance. The amplitude of the incident light can be significantly enhanced via excitation of a surface plasmon enhancing the luminescence response. However, when the resonance frequency of the metal nanostructure corresponds to the frequency of the emitters, their energy absorption can be greatly enhanced. The final mechanism envisions the case in which the resonance frequency of the metal nanostructure corresponds to the emission frequency of the emitter, in which case the radiative decay-rate increases for fluorophores with an intrinsic quantum yield less than one.

**Figure 10:**
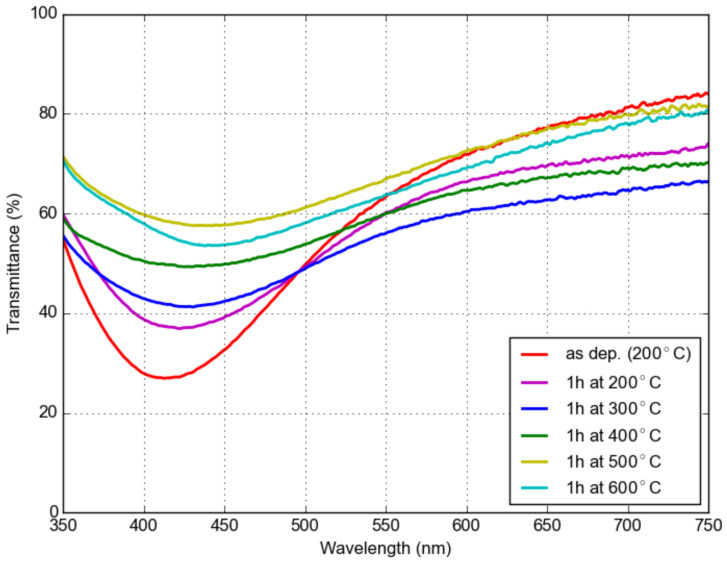
Transmittance of a layer of LaF_3_ doped with 7 at.% Yb^3+^ and 1 at.% Er^3+^ with dispersed Ag nanoparticles on a fused silica substrate annealed at various temperatures.

**Figure 11:**
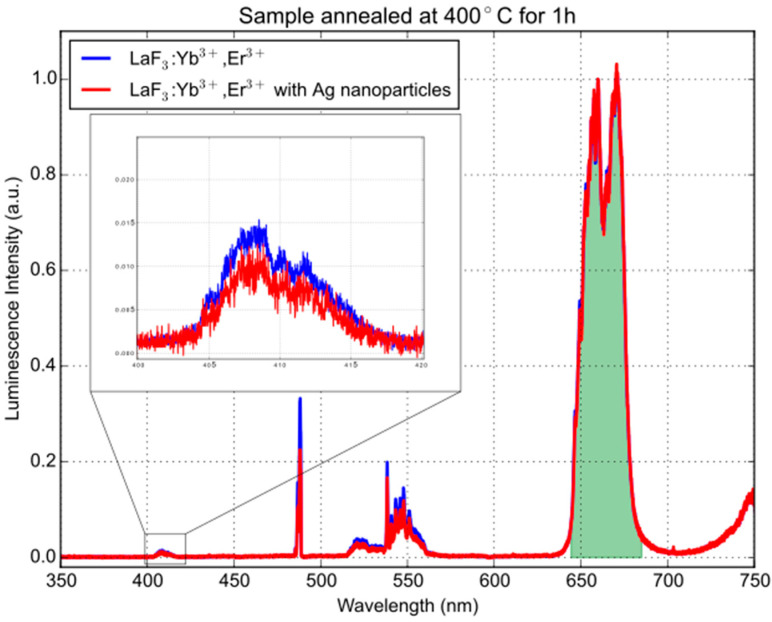
The up-conversion emission spectrum of the sample annealed at 400 °C excited by a 200 mW 980 nm diode laser. The intensity is normalized to the peak indicated by the green area.

Figure 10 depicts the transmittance spectra of the LaF_3_ coating doped with 7 at.% Yb^3+^ and 1 at.% Er^3+^ with dispersed Ag nanoparticles on a fused silica substrate. The sample was annealed at various temperatures ranging from 200 °C to 600 °C for 1 h. The non-annealed sample exhibited a broad absorption peak around 400 nm. This absorption peak shifted to longer wavelengths after annealing. The up-conversion emission spectrum of the sample annealed at 400 °C is shown in Figure 11. The emission was excited using a 200 mW 980 nm diode laser. The sharp peak at 488 nm is a second harmonic of the laser. The intensity is normalized to the most intense peak above 650 nm (green area). The Er^3+^ and Yb^3+^ ion pair has one emission peak at 407 nm. This wavelength overlaps with the SPR of Ag NPs, making it possible to study the effect of metal-enhanced fluorescence (MEF) of the Ag NPs by comparing the spectra for layers with and without Ag NPs. The inset shows the emission peak that corresponds to the resonance frequency of the Ag NPs. We can observe a slight reduction in the emission for the coating with Ag NPs.

**Figure 12:**
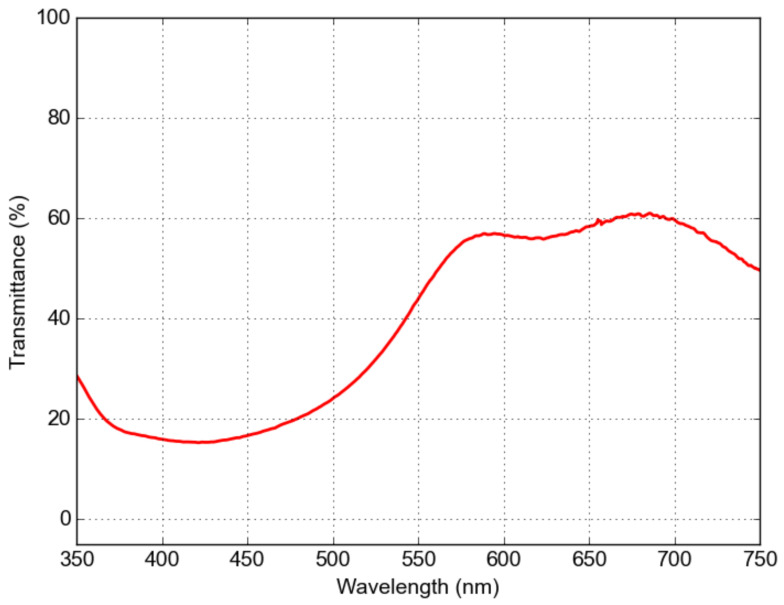
Transmission spectrum of Eu^3+^-doped (15%) LaF_3_ nanocomposite LaF_3_.

**Figure 13:**
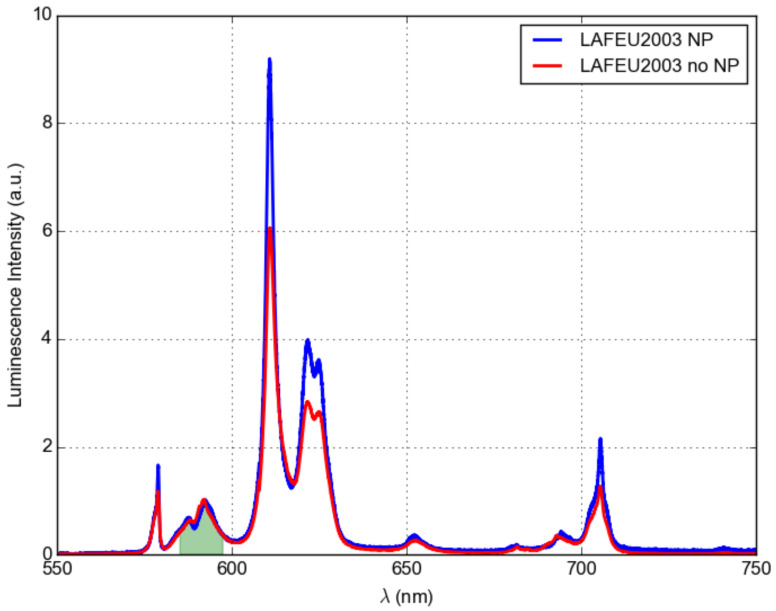
Luminesce spectrum of Eu^3+^-doped (15%) LaF_3_ nanocomposite LaF_3_. The intensity is normalized to the peak indicated by the green area.

The transmission spectrum of the Eu^3+^-doped LaF_3_ nanocomposite shows a broad absorption peak at approximately 400–450 nm, as shown in the transmittance spectra in Figure 12. The emission spectra shown in Figure 13 were excited using a 100 mW laser diode at 403 nm. The emission at approximately 590 nm corresponds to the ^5^D_0_ → ^7^F_1_ magnetic dipole (MD) transition, while the dominant bands around 620 nm, 650 nm, and 690 nm correspond to ^5^D_0_ → ^7^F_2,3,4_ electric dipole (ED) transitions. One can observe a distinctly higher ED emission for the sample with Ag NPs. There is a significant quenching effect in the vicinity of the metal surface that is different for ED and MD transitions [32].

## 4. Conclusions

AFM measurement of NPs deposited on a silicon substrate for various distances between the magnetron and the orifice indicates that mean particle size increases with distance. Sizes range from about 3.6 nm for 50 mm to about 8.4 nm for 150 mm. Size saturates at a distance of 150 mm and remains approximately constant up to 250 mm. The FWHM of the size distribution increases with distance as well. Working gas pressure and magnetron power do not significantly affect particle size. STEM images confirm the spherical shape of larger NPs and their polycrystalline nature. Ellipsometry measurement shows an SPR peak in the extinction coefficient at a wavelength of 380 nm. Finally, we tested the effect of Ag NPs on lanthanide ions for downshifting and up-conversion photoemission nanocomposites.

## Data Availability

Data will be available upon reasonable request from the corresponding author.
